# Carboplatin plus Paclitaxel in Combination with the Histone Deacetylate Inhibitor, Vorinostat, in Patients with Recurrent Platinum-Sensitive Ovarian Cancer

**DOI:** 10.3390/jcm13030897

**Published:** 2024-02-03

**Authors:** Hanieh Meteran, Anja Ør Knudsen, Trine Lembrecht Jørgensen, Dorte Nielsen, Jørn Herrstedt

**Affiliations:** 1 Department of Clinical Oncology and Palliative Care, Zealand University Hospital, 4000 Roskilde, Denmark; hanieh.meteran@gmail.com; 2 Department of Oncology, Odense University Hospital, 5000 Odense, Denmark; anja.oer.knudsen@rsyd.dk (A.Ø.K.); trine.joergensen@rsyd.dk (T.L.J.); 3 Institute of Clinical Research, University of Southern Denmark, 5230 Odense, Denmark; 4 Department of Oncology, Copenhagen University Hospital, Herlev and Gentofte Hospital, 2730 Copenhagen, Denmark; dorte.nielsen.01@regionh.dk; 5 Department of Clinical Medicine, Faculty of Health and Medical Sciences, University of Copenhagen, 2200 Copenhagen, Denmark

**Keywords:** ovarian cancer, platinum-sensitive recurrence, histone deacetylase inhibitor, vorinostat

## Abstract

**Background:** This phase II study evaluated the efficacy and safety of the histone deacetylase (HDAC) inhibitor, vorinostat, administered in combination with paclitaxel and carboplatin in patients with platinum sensitive recurrent ovarian cancer. **Methods:** Women with recurrent platinum-sensitive ovarian, peritoneal, or Fallopian tube carcinoma, a performance status of 0–2, and good overall organ function were eligible. Patients received 6 courses of paclitaxel (175 mg/m^2^) and carboplatin area under the curve (AUC) of 5.0 mg/mL/min administered via intravenous infusion on day 1 of a 3-week schedule. In addition, patients received vorinostat 400 mg orally once daily on days −4 through 10 of Cycle 1 and days 1 through 14 of each subsequent treatment cycle. The primary endpoints were progression-free survival (PFS) and adverse events. The secondary endpoints were the objective response rate and overall survival. **Results:** Fifty-five patients were included. CR was obtained in 14 patients (26.4%) and PR in 19 patients (35.8%), resulting in an ORR of 62.2%. Twenty patients (37.7%) had SD. The median duration of response (DoR) was 12.6 (range 6–128) months. The median PFS was 11.6 months (95% CI, 10.3–18.0; *p* < 0.001). Median OS was 40.6 months (95% Cl, 25.1–56.1). The most common treatment-related adverse events (all grades) were fatigue, anemia, thrombocytopenia, neutropenia, anorexia, nausea, pain, sensory neuropathy, myalgia, stomatitis and diarrhea. **Conclusions:** Vorinostat combined with carboplatin plus paclitaxel was tolerable and generated significant responses including a long median overall survival in recurrent platinum-sensitive ovarian cancer.

## 1. Introduction

Epithelial ovarian cancer (EOC) is the fifth most common cancer in women and the leading cause of gynecologic cancer death worldwide [1]. Due to the absence of symptoms in the early stages of the disease and the lack of effective screening methods, most patients have advanced-stage disease (III–IV) at the time of diagnosis [2,3], challenging the efficacy of treatments. As many as 75% of patients with stage III-IV ovarian cancer will experience relapse after standard treatment [4], and each subsequent recurrence occurs in shorter intervals until chemotherapy resistance develops [5]. Consequently, the 5-year overall survival rate is approximately 30–50% [6].

The evidence-based, guideline-recommended standard treatment for advanced stage EOC consists of optimal cytoreductive surgery followed by 3-weekly carboplatin plus paclitaxel [7]. Efforts have been made to improve the standard two-drug regimen by adding a third cytotoxic agent; however, the results only demonstrated increased toxicity and failed to improve progression-free survival or overall survival [8,9]. Carboplatin-based doublet is also the first choice in the second-line treatment of platinum-sensitive patients, while agents like liposomal doxorubicin, topotecan, weekly paclitaxel, or gemcitabine given as a single agent are used in platinum-resistant patients [7].

While there have been great advances of novel agents for the treatment of many solid malignancies, the progress in the treatment of ovarian cancer has generally lagged behind. However, over the past several years, targeted therapies have emerged and been included in the treatment of EOC [10]. Bevacizumab, an anti-angiogenesis inhibitor, and poly-ADP-ribose polymerase (PARP) inhibitors (olaparib, rucaparib, and niraparib) have been approved for first-line and platinum-sensitive recurrent maintenance therapy, improving clinical outcomes in terms of progression-free survival [10,11,12]. PARP inhibitors have the greatest benefit in BRCA 1/2 mutated tumors, but are also superior to placebo in BRCA 1–2 wild type high-grade serous or endometrial adenocarcinoma [13]. Despite the advancements in the treatment of EOC, the long-term prognosis remains poor, stressing the need for continued exploration of novel therapies to augment the efficacy of current treatments and further enhance patient outcomes.

Given the importance of epigenetic alterations in the development of cancer, epigenetic-modifying enzymes are emerging as potential targets for the treatment of cancer [14]. Histone deacetylases (HDACs) are a class of enzymes that modifies gene expression by altering the acetylation status of nucleosomal histones and non-histone proteins. While increased acetylation of histones opens the DNA to transcription factors that promote gene expression, HDACs downregulate the expression of genes, including tumor suppressor genes [15]. Thus, targeting the HDACs result in the accumulation of acetylated proteins inducing multiple cellular anti-tumor effects. Vorinostat is a potent inhibitor of HDAC that has been shown to inhibit cell growth and cell differentiation, and to induce apoptosis of tumor cells [16]. In 2006, vorinostat was approved by the FDA for the treatment of cutaneous T-cell lymphoma and was also active in other hematological malignancies [16]. Clinical trials with HDAC inhibitors in the treatment of ovarian cancer demonstrated only modest effects [17,18,19,20]. To date, however, the number of clinical studies has been limited, while there is mounting preclinical data supporting the synergistic cytotoxic effects of HDAC inhibitors and conventional chemotherapy [21,22,23]. The combination of vorinostat and carboplatin plus paclitaxel was evaluated in a phase I study of patients with advanced solid malignancies [24]. The dose-schedule of vorinostat in our study was established by this phase I study, in which vorinostat was administered in doses up to 400 mg o.d for 14 days in combination with carboplatin (area under the curve defined as 6.0 mg/mL/min) and paclitaxel (200 mg/m^2^) without dose-limiting toxicity. Dose-limiting toxicities (in higher doses) included grade 3 emesis and grade 4 neutropenia with fever, while non-limiting toxicity included nausea, diarrhea, fatigue, neuropathy, thrombocytopenia, and anemia.

This phase II study was designed to evaluate the efficacy and safety of vorinostat administered in combination with paclitaxel and carboplatin in patients with advanced recurrent platinum-sensitive EOC.

## 2. Materials and Methods

### 2.1. Design and Objectives

In the period 2007–2012, a phase II study of the effect and adverse events of carboplatin plus paclitaxel in combination with vorinostat (suberoylanilide hydroxamic acid, SAHA) in women with recurrent platinum-sensitive EOC, Fallopian tube cancer, or primary peritoneal carcinoma (hereafter referred to as ovarian cancer) was conducted. The prospective phase II study was performed at the Department of Oncology, Odense University Hospital, and at the Department of Oncology, Herlev Hospital. Patients were treated with a maximum of 6 cycles or until disease progression, unacceptable toxicity, or withdrawal of consent occurred. Written informed consent was obtained from all patients prior to study entry. The study was conducted in accordance with Good Clinical Practice and the Declaration of Helsinki and was approved by the Ethics Committee (H-KA-20070007) and by the Danish Health Authorities (2612-3399). This study was registered on ClinicalTrials.gov under the identifier NCT00772798 (Eudract 2006-007013-20). The study was closed in 2012. The current publication is based on the original data (prospectively collected 2007–2012) and on a retrospective follow up from 2012 until June 2023.

The primary objective was to evaluate the efficacy, based on progression-free survival (PFS), and to examine the adverse events of vorinostat in combination with carboplatin plus paclitaxel. Secondary objectives were the objective response rate (ORR), overall survival (OS), and the comparison of PFS of the first-line treatment with the second-line treatment.

### 2.2. Patients

Inclusion and exclusion criteria for enrollment in the phase II study are defined in Table 1. Briefly, eligible patients were females ≥ 18 years with a histologically confirmed diagnosis of EOC. All patients had received first-line therapy with carboplatin plus paclitaxel. Patients had to be platinum-sensitive, defined as the recurrence or progression of ovarian cancer 6 months or later after the end of first-line chemotherapy. Patients were required to have a performance status of 0–2, acceptable renal and hepatic function, and be free of other serious illness that could impair their ability to receive the protocol-defined therapy (Table 1).

### 2.3. Treatment Plan

Eligible patients were to receive 6 courses of paclitaxel (175 mg/m^2^) and carboplatin AUC 5 (area under the curve defined as 5.0 mg/mL/min) administered via intravenous infusion (IV) on day 1 of a 3-weekly schedule, which was the recommended standard treatment in 2007 [25].

In addition, all eligible patients received vorinostat 400 mg administered orally once daily on days −4 through 10 of Cycle 1 (25-day treatment cycle) and days 1 through 14 of each subsequent 21-day treatment cycle. Patients received antiemetic therapy according to the institutional guidelines as well as premedication with dexamethasone and antihistamines (an H_1_-receptor antagonist and an H_2_-receptor antagonist) for prevention of hypersensitivity induced by paclitaxel.

### 2.4. Assessments and Endpoints

Patient evaluation included patient history, physical examination, laboratory assessments including CA-125, urine analysis, electrocardiogram (ECG), and computed tomography (CT) of the chest and abdomen. Physical examination, CA-125 measurement, and urine analysis was performed pre-treatment and on day 1 of each cycle. ECGs were obtained 2 h after treatment with vorinostat on day −4 in cycle 1 as well as weekly. Laboratory assessments, which included complete blood count, liver enzymes, clotting screen, creatinine, electrolytes, glucose, and uric acid, were performed as pre-treatment and weekly during cycle 1 and as pretreatment during cycle 2–6. Treatment response was assessed after 3 and 6 cycles using computed tomography (CT) of the chest and abdomen. In patients with stable disease or response to treatment, CT was performed every 6 weeks for the first 3 months after the end of treatment, and subsequently every 12 weeks until disease progression or the end of study.

The primary endpoints were progression-free survival (PFS) and adverse events. The secondary endpoints were the objective response rate (ORR) and overall survival (OS). Comparison of PFS of the first-line treatment with the second-line treatment was an exploratory endpoint. PFS was defined as the time from the initial treatment to the first recurrence or death from any cause or the last follow up. OS was defined as the time interval between the date from the initial treatment to death from any cause or the last follow up. Progression could be based on radiological assessment or the evaluation of serum CA-125. While serum CA-125 progression was defined per the Gynecological Cancer Intergroup progression definition [26], ORR and radiological progression were defined according to the Response Evaluation Criteria in Solid Tumors (RECIST version 1.0) [27]. The RECIST 1.0 assessments were performed by a specialist in radiology. The safeness of the treatment was determined by assessing the frequency and intensity of adverse events as defined by the National Cancer Institute Common Terminology Criteria for Adverse Events version 3 (CTCAEv.3). Toxicity evaluations were performed at baseline and before every treatment cycle throughout the study. Patients with adverse events were followed until a return to baseline or ≤grade 1 occurred.

### 2.5. Statistics

This is a non-blinded, non-comparative study using descriptive statistics. For the comparison between 1. line and 2. line PFS (exploratory endpoint), we used the Wilcoxon matched-pairs rank sum test.

*p* < 0.05 was considered statistically significant. PFS and OS were summarized using the Kaplan–Meier method. Confidence intervals were included where appropriate. The statistical software SPSS version 28.0 (SPSS Inc., Chicago, IL, USA) was used for the statistical analyses. The study was initially planned to include 35 patients, but was amended to include 55 patients.

## 3. Results

Fifty-five patients with advanced, recurrent ovarian, Fallopian tube, or primary peritoneal cancer were enrolled between June 2007 and May 2012. Patient demographics and baseline disease characteristics are summarized in Table 2. The BRCA status of the patients was unknown. Since all patients (except for two patients still alive) were followed until death, the median follow up is identical with the median OS (40.6 months with the longest follow up of 15 years).

In first-line carboplatin + paclitaxel, forty patients (72.7%) had completed 6 cycles; 3 patients (5.5%), 7 cycles; 2 patients (3.6%), 9 cycles; and one patient (1.8%), 5 cycles. The remaining 9 patients had completed 4–6 cycles, but the exact number was not available (16.4%). In the current study, patients received a median number of 5 cycles of vorinostat, carboplatin, and paclitaxel (range 1–6). All patients, with the exception of two patients, were followed until death.

### 3.1. Response

Of the 55 patients, 53 patients (96.4%) were assessable for response evaluation. One patient had no response evaluation and one patient was lost to follow up.

The best overall response was CR for 14 patients (26.4%) and PR for 19 patients (35.8%), resulting in a total best overall response rate of 62.2%. Twenty patients (37.7%) had SD. The best overall responses in the first-line treatment and in the present study are presented in Table 2. The median duration of response (DoR) for patients with CR and PR in the second-line treatment was 12.6 (range 6–144) months. The response duration as compared to the response duration in first-line therapy is reported in Table 3. Seven patients (21%) had a longer response duration after the second-line treatment as compared to the first-line treatment and an additional two patients had a longer duration of stable disease.

### 3.2. Progression-Free and Overall Survival

**Figure 1:**
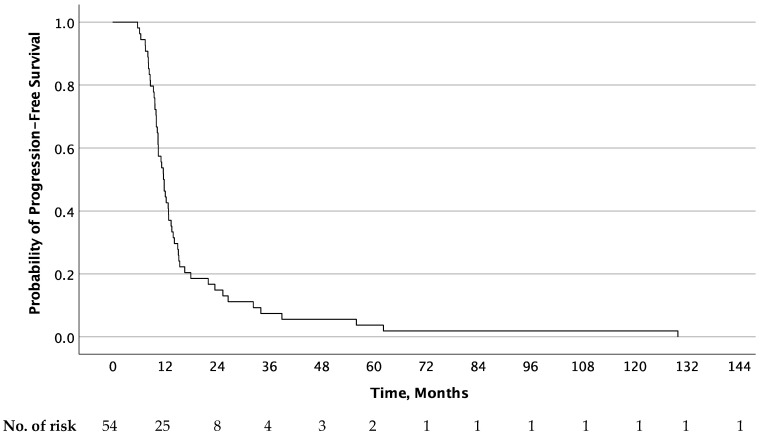
Progression-free survival after second-line treatment (carboplatin, paclitaxel, and vorinostat).

**Figure 2:**
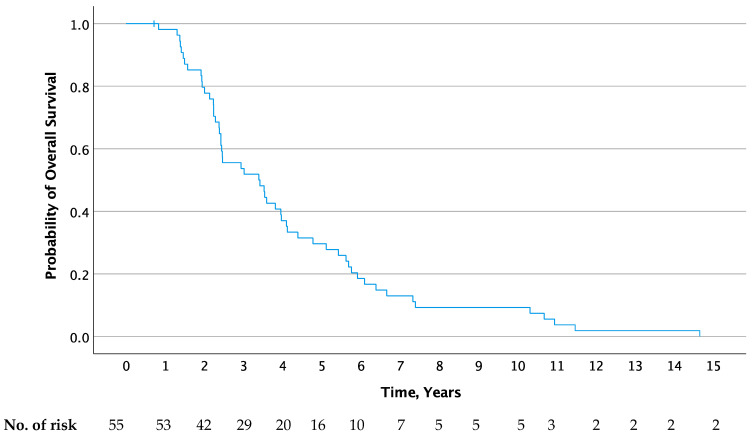
Overall survival after second-line treatment (carboplatin, paclitaxel, and vorinostat).

All 55 patients were assessable for overall survival analysis, while 54 patients were assessable for analysis of PFS. The Kaplan–Meier curves for PFS of the second-line treatment as well as OS are shown in Figure 1 and Figure 2, respectively. The median PFS of the first-line treatment was significantly longer than that reached in the second-line treatment (15.6 months; 95% CI, 13.6–18.8 vs. 11.6 months; 95% CI, 10.3–18.0; *p* < 0.001). At one year, 80% (95% Cl, 67–90) and 45.5% (95% Cl, 32–59) of patients were progression-free in the first- and second-line treatment, respectively. Median OS was 40.6 months (95% Cl, 25.1–56.1) after the start of second-line therapy. At the end of follow up (cutoff 1 June 2023), two patients were still alive 15.6 and 11.7 years after study inclusion, one of whom was still without progressive disease more than 10 years after the end of treatment.

### 3.3. Toxicity

Although the median number of cycles completed was five, twenty-nine patients (52.7%) discontinued treatment before the completion of six cycles. Reasons for the early discontinuation were adverse events (17), patient request (9), problems with the delivery of vorinostat (2), and surgery for metastatic disease (1).

The reasons patients requested to discontinue treatment were fatigue for two of the patients, and the remaining were (1) vomiting; (2) nausea, arthralgia, and motor neuropathy; (3) fatigue, diarrhea, and anorexia; (4) chest tightness; (5) fatigue, constipation, pain, taste alterations, and stomatitis; and (6) neutro-thrombocytopenia. The last patient requested to discontinue treatment in order to go on vacation.

Dose reduction was required in 10 patients (18%) and treatment delay of at least one cycle was necessary in 24 patients (43.6%). Treatment-related adverse events occurring in ≥ 10% of patients are shown in Table 4. The most common treatment-related adverse events (all grades) were fatigue (91%), anemia (90%), thrombocytopenia (86%), neutropenia (84%), anorexia (80%), nausea (78%), pain (76%), sensory neuropathy (75%), myalgia (62%), stomatitis (55%), and diarrhea (51%). An overview of grade 1–4 adverse events is shown in Table 4. Grade 4 adverse events were limited to neutropenia (16%), thrombocytopenia (4%), and fatigue, sensory neuropathy, and pain (each 1.8%). Of note, three patients (5.5%) experienced febrile neutropenia. No grade 5 events were reported. One patient had QTc prolongation (492 ms) 2 h after administration of the first dose of vorinostat. The patient had no symptoms and was clinically unaffected; however, the event was reported as a serious adverse event and led to discontinuation of the study treatment.

## 4. Discussion

This phase II study was designed to evaluate the efficacy and safety of the pan-HDAC inhibitor, vorinostat, administered in combination with paclitaxel and carboplatin for the treatment of advanced recurrent, platinum-sensitive ovarian cancer. The study was conducted before targeted therapy became standard in combination with platinum-based chemotherapy in platinum-sensitive recurrent disease. The most remarkable finding was a median OS of 40.6 months, including two patients who were still alive more than 11 and 15 years after the end of treatment, respectively.

Our response rate of 62% and median PFS of 11.6 months are comparable with that of second-line carboplatin and paclitaxel alone [28], and similar to the response rate of 63% obtained in a phase II study of another HDAC inhibitor, belinostat, in combination with carboplatin and paclitaxel [20]. However, the response rate was significantly higher than in the trials investigating HDAC inhibitors alone, with response rates ranging from 0 to 4% [17,18]. While our study revealed a relatively high response rate, this finding did not translate into a superior PFS as compared with PFS in the first-line treatment. In contrast, the median PFS of vorinostat in combination with paclitaxel and carboplatin was significantly shorter than that reached in the first-line treatment. This is in line with the results from other studies [5]. However, our study revealed a response duration of 12.6 months, which is an underutilized clinical endpoint in this field of research, although highly important in terms of the clinical benefit patients may obtain from treatments [29]. Only one other HDAC inhibitor study utilized the duration of response, which was 5.8 months, though the best overall response in that study was SD [18]. It is also noteworthy that in our study, seven patients (21%) had a longer response duration after the second-line treatment as compared to the first-line treatment. In a retrospective study (*n* = 211), patients with platinum-sensitive recurrent ovarian cancer were receiving platinum-based chemotherapy in the 2. line. Only 4 out of 211 had a longer response duration in the 2. line and three of these patients did not receive a taxane in the 1. line [5].

Of note, the OS of 40.6 months observed in this study is longer than expected in second-line chemotherapy alone, including carboplatin and paclitaxel (29 months) [28], carboplatin and pegylated liposomal doxorubicin (31 months) [30], and carboplatin and gemcitabine (18 months) [31]. To date, only one other phase II study examined HDAC inhibitors in patients with platinum-sensitive ovarian cancer; however, OS was not reached during study follow up [20]. The OS benefit in our study could be influenced by subsequent lines of treatment; however, it could also be hypothesized that vorinostat increases the effect of subsequent treatments in specific patients.

To date, there have only been five phase II studies on HDAC inhibitors in the treatment of ovarian cancer: two and three, respectively, investigating vorinostat and belinostat [17,18,19,20,32]. Although direct comparison might be challenging due to heterogeneity across study designs and patient populations, there seems to be a pattern with regards to clinical activity with limited efficacy of HDAC inhibitors as a single agent [17,18] and modest efficacy in combination with chemotherapy [19,20]. The exact molecular pathway of HDAC inhibitors is not fully understood. Since the preclinical data demonstrated that HDAC inhibition exert a synergistic therapeutic effect via its combination with cytotoxic chemotherapies such as carboplatin and paclitaxel [21,33], HDAC inhibitors have primarily been used concomitantly with chemotherapy without subsequent maintenance HDAC inhibitor treatment.

With respect to safety, the most common grade 3 and 4 toxicities were hematologic, including neutropenia and thrombocytopenia. As expected, the three-drug combination regimen was associated with increased toxicity compared to carboplatin and paclitaxel alone, as well as fewer patients completing six courses of chemotherapy [28]. Compared to other studies of vorinostat in combination with chemotherapy, the adverse events in this study did seem relatively manageable. Other studies revealed extensive hematologic toxicities [32], including a phase I study by Matulonis et al. [34] investigating vorinostat in combination with carboplatin and gemcitabine. The investigators performed dose escalation of vorinostat up to 400 mg daily. Due to grade 3–4 neutropenia and thrombocytopenia of 60% and 53%, respectively, the study was terminated ahead of time despite achieving PR in six out of seven patients.

Some limitations of the study should be emphasized. The study was non-comparative including a limited number of patients. No molecular characterization of the patients or the tumors was performed. It is a strength that all patients were followed until death, meaning that all events of PFS was reported (*n* = 53) and all (but two) events of OS (*n* = 53) was reported. This also means that the continuous debate about using PFS or OS as the primary effect parameter [35] is not an issue in this study.

Since the initiation of the current study, the treatment of ovarian cancer has evolved greatly with approval of bevacizumab and PARP inhibitors. In 2014, bevacizumab, a monoclonal antibody targeting the vascular endothelial growth factor, was approved by EMA in combination with chemotherapy for platinum-sensitive recurrent EOC. Bevacizumab plus carboplatin and gemcitabine improved PFS compared to the placebo plus carboplatin and gemcitabine (12.4 vs. 8.4 months), but no significant difference in OS was observed [36]. Our PFS is comparable with that of bevacizumab in addition to chemotherapy, but we had a notably longer OS (40.6 vs. 33.6 months) and DoR (12.6 vs. 10.4 months), underlining the promising potential of vorinostat in patients with platinum-sensitive recurrent EOC. The PARP inhibitors olaparib, niraparib, and rucaparib led to a significant PFS benefit in patients with homologous recombination deficiency (HRD) or BRCA-mutated ovarian cancer previously treated with and responding to platinum-based regimens [37,38,39], and in two studies in BRCA mutated patients (one in first line and one in platinum-sensitive recurrent disease), it nearly resulted in a statistically significant prolonged OS [39,40]. The NOVA study examining niraparib found the largest improvement of PFS in BRCA-mutated patients, but also prolonged PFS in BRCA wild type patients [38].

In the field of histone modification for the treatment of ovarian cancer, future perspectives include not only combinations with chemotherapy, but also HDAC inhibitors in combination with other targeted therapies. Indeed, panobinostat, another pan-HDAC inhibitor similar to vorinostat, has shown to enhance olaparib efficacy by reducing cell proliferation, increasing DNA damage, as well as T-cell infiltration, and ultimately reducing peritoneal masses and tumor burden in a mouse model of ovarian cancer [41].

Another aspect to consider is the development and use of selective HDAC inhibitors. While the majority of HDAC inhibitors tested are pan-HDAC inhibitors acting on several protein isoforms, this may not be the most efficient strategy. While normal ovarian epithelium displays weak nuclear expression of class I HDAC, an increased expression of class I HDAC, including HDAC 1, 2, and 3, has been reported in ovarian cancer [22]. Not all HDAC isoforms are, however, abnormally expressed in all types of cancer [42]. Class I HDAC inhibitors, specifically HDAC2, is abnormally expressed in several cancers including ovarian [43]. A higher expression of HDAC2 is also found to be an important marker of poor prognostic factors in different types of cancer [42]. Second, the use of pan-HDAC inhibitors can have many off-target effects resulting in severe adverse events. Thus, selective HDAC inhibitors might represent novel targets of investigation for the treatment of recurrent ovarian cancer.

## 5. Conclusions

Treatment with vorinostat in combination with carboplatin and paclitaxel was tolerable and generated significant response in patients with platinum-sensitive recurrent EOC. While the PFS in our study was comparable to that of carboplatin and paclitaxel alone in the recurrent setting, it demonstrated a long response duration and an impressive and competitive overall survival. Taken together, this study warrants further investigation of HDAC inhibitors in combination with chemotherapy in this patient population.

## Figures and Tables

**Table 1:** Inclusion and exclusion criteria.

Inclusion Criteria	Exclusion Criteria
Histological-verified epithelial ovarian, peritoneal, or fallopian tube carcinoma.	Patients treated with an experimental drug within the last 4 weeks before inclusion, and patients who received other concomitant anticancer treatments.
Women ≥ 18 years.	Patients having an active infection or who have received intravenous antibacterial or antifungal medicine within the last 2 weeks before inclusion.
ECOG performance status ≤ 2.	Previous treatment with more than first-line chemotherapy.
Expected duration of life > 3 months.	Patients previously treated with an HDAC inhibitor. Patients who have been treated with Valproate for convulsions can be included; however, only if the treatment has taken place > 30 days before inclusion.
Previous treatment regimen containing platinum and paclitaxel.	Patients treated with steroid who are not stabilized on a firm dose equivalent to a maximum of 10 mg prednisolone per day for the last 4 weeks before inclusion.
Platinum and paclitaxel sensitive tumor, defined as a minimum of 6 months from cessation of treatment until disease progression.	Progression during treatment with first-line chemotherapy containing platinum/paclitaxel or disease progression less than 6 months after treatment cessation.
Measurable or assessable lesion. Patients having increased CA-125 as the only sign of recurrence are also eligible.	Concomitant serious and/or non-controllable medical condition such as non-controllable infection (including HIV-infected patients), hypertension, ischemic heart disease, myocardial infarction within the last 6 months, or congestive heart failure.
Signed informed consent before inclusion.	Previous treatment for or another concomitant malignant disease within the last 5 years, except for curative-treated carcinoma in situ cervical cancer, or basal cell carcinoma.
Prepared to appear for the planned follow-up visits and capable of handling toxicity.	Previous severe allergic reactions in connection with carboplatin, paclitaxel, or agents within the histone deacetylase inhibitor group.
Normal organ functions *	Women of child-bearing age. Women must have undergone surgical removal of the ovaries or be post-menopausal with no menstruation during the previous year.
	Peripheral neuropathy ≥ grade 2, unless this is due to a medical condition.
	Patients with history of severe hypersensitive reactions with regards to products containing cyclosporine or K-vitamin) and/or patients with known hypersensitivity towards agents chemically connected to paclitaxel, carboplatin, or vorinostat.
	Patients with known cerebral metastases or clinical signs of cerebral metastases.

* Defined by the following values: Absolute neutrophil count ≥ 1500/μL; platelets ≥ 100,000/μL; hemoglobin ≥ 9.0 g/dL or >5.7 mmol/L; CA125 0–35; glomerular filtration rate (GFR) measured using Cr-EDTA clearance ≥ 50 mL/minute; serum total bilirubin ≤ 1.5 times the upper level of normal (ULN); AST (SGOT) and ALT (SGPT) ≤ 2.5 times the ULN; alkaline phosphatase ≤ 5.0 times the ULN; prothrombin time (PT) ≤ 1.2 times the ULN unless the patient is receiving therapeutic anticoagulation; partial thromboplastin time (PTT) ≤ 1.2 times the ULN unless the patient is receiving therapeutic anticoagulation.

**Table 2:** Patient demographics and disease characteristics.

	*N* = 55
Age, median (range)	62 (44–78)
ECOG performance status, *n* (%)	
0	49 (89.1)
1	5 (9.0)
2	1 (1.8)
Malignancy, *n* (%)	
Ovarian	43 (72.7)
Primary peritoneal carcinoma	10 (18.1)
Fallopian tube carcinoma	2 (3.6)
Histologic subtype and tumor grade, *n* (%)	
Serous	
High grade	28 (50.9)
Intermediate	10 (18.2)
Low grade	5 (9.1)
Unknown	7 (12.7)
Endometrioid	
High grade	1 (1.8)
Unknown	1 (1.8)
Clear cell	1 (1.8)
Mixed endometrioid/serous	
High grade	1 (1.8)
Transitional cell	
High grade	1 (1.8)
Platinum-free interval, months	
6–12	10 (18.2)
>12–18	22 (40.0)
>18–24	8 (14.5)
>24	15 (27.3)

ECOG, Eastern Cooperative Oncology Group.

**Table 3:** Response rates and duration of responses.

Response	First-Line Treatment *n* (%)	Second-Line Treatment *n* (%)	Duration of Responses (Months)	First-Line *n* = 45	Second-Line *n* = 33	Secondary Response Rates Based on Duration of First-Line Response *n* = 29 (%)
**CR**	32 (58.1)	14 (25.5)	**<6**		1	
**PR**	13 (23.6)	19 (34.5)	**6–12**	6	14	3 (50)
**SD**	7 (12.7)	20 (36.4)	**>12–18**	21	12	13 (62)
**Unknown**	3 (5.5)	2 (3.6)	**>18–24**	6	2	4 (67)
			**>24**	12	4	9 (75)

**Table 4:** Treatment-related adverse events.

Adverse Events	Any Grade *n* (%)	Grade 1–2 *n* (%)	Grade 3–4 *n* (%)
*Non-haematological (patients, n = 55)*			
Diarrhea	28 (50.9)	25 (45.5)	3 (5.5)
Nausea	45 (81.8)	44 (80.0)	1 (1.8)
Vomiting	27 (49.1)	25 (45.5)	2 (3.6)
Dyspepsia	7 (12.7)	7 (12.7)	0 (0)
Constipation	22 (40.0)	20 (36.4)	2 (3.6)
Cystitis	9 (16.4)	9 (16.4)	0 (0)
Sensory neuropathy	41 (74.5)	39 (70.9)	2 (3.6)
Motor	22 (40.0)	20 (36.4)	2 (3.6)
Pain	42 (76.3)	39 (70.9)	3 (5.5)
Stomatitis	30 (54.5)	30 (54.5)	0 (0)
Skin	17 (30.9)	17 (30.9)	0 (0)
Nail disorder	7 (12.7)	6 (10.9)	1 (1.8)
Allergic reaction	7 (12.7)	5 (9.1)	2 (3.6)
Dyspnea	15 (27.3)	14 (25.5)	1 (1.8)
Fever in absence of infection	8 (14.5)	8 (14.5)	0 (0)
Fatigue	50 (90.9)	36 (65.5)	14 (25.5)
Anorexia	44 (80.0)	41 (74.5)	3 (5.5)
Myalgia	34 (61.8)	33 (60)	1 (1.8)
Arthralgia	27 (49.1)	27 (49.1)	0 (0)
*Haematological (patients, n = 50)*			
Neutropenia ^1^	42 (84)	15 (30)	27 (54)
Thrombocytopenia ^2^	43 (86)	33 (66)	10 (20)
Anemia ^3^	45 (90)	45 (90)	0 (0)
Increased creatinine	5 (10)	5 (10)	0 (0)

^1^: The category of neutropenia includes reports of neutropenia and decreased neutrophil count. ^2^: The category of thrombocytopenia includes reports of thrombocytopenia and decreased platelet count. ^3^: The category of anemia includes reports of anemia and decreased hemoglobin count.

## Data Availability

Dataset available on the request to the corresponding author.
